# Genetic Approaches for Controlling CRISPR-based Autonomous Homing Gene Drives

**DOI:** 10.3389/fbioe.2022.897231

**Published:** 2022-06-15

**Authors:** Pratima R. Chennuri, Zach N. Adelman, Kevin M. Myles

**Affiliations:** Department of Entomology, Texas A & M University, Minnie Belle Heep Center, College Station, TX, United States

**Keywords:** CRISPR, Cas9, autonomous, homing, gene drive, controlling systems, braking systems

## Abstract

CRISPR-based autonomous homing gene drives are a potentially transformative technology with the power to reduce the prevalence of, or even eliminate, vector-borne diseases, agricultural pests, and invasive species. However, there are a number of regulatory, ethical, environmental, and sociopolitical concerns surrounding the potential use of gene drives, particularly regarding the possibility for any unintended outcomes that might result from such a powerful technology. Therefore, there is an imminent need for countermeasures or technologies capable of exerting precise spatiotemporal control of gene drives, if their transformative potential is ever to be fully realized. This review summarizes the current state of the art in the development of technologies to prevent the uncontrolled spread of CRISPR-based autonomous homing gene drives.

## 1 Introduction

The ability to autonomously drive a trait of interest through a natural population in order to effect genetic control is a long sought-after goal. Early attempts at realizing this goal focused on the co-option of site-specific homing endonuclease genes [HEGs; (Burt, 2003, 2014; Windbichler et al., 2007, 2011; Chan et al., 2011)]. HEGs are naturally occurring selfish genetic elements that encode a nuclease, which recognizes and cleaves a 15–30 bp sequence that typically occurs only once in the genome. HEGs occurring in the middle of their own recognition site can then be used as a repair template and copied over into the cleaved site through the cell’s DNA repair processes (Burt and Koufopanou, 2004). However, HEGs are constrained by highly specific protein-DNA interactions, which restricted design choices and limited their applicability to target new locations. The discovery of CRISPR-based genome editing opened the door to the development of more facile and powerful genome engineering tools by making nearly every nucleotide sequence in the genome accessible to editing (Doudna and Charpentier, 2014). As a result, since 2015, the development of CRISPR-based autonomous drives has progressed rapidly in both model and non-model organisms (Gantz and Bier, 2016; Hammond et al., 2016; Kyrou et al., 2018; Oberhofer et al., 2019; Simoni et al., 2020).

A major hurdle arising early in the development of CRISPR-based autonomous drives was the appearance of target alleles that are refractory to further Cas9 cleavage. These refractory target sites, termed “resistant” alleles, occurred as a consequence of either existing genetic variation, *de novo* mutation, or erroneous repair of the DNA double stranded breaks (DSBs) created by Cas9. In any case, the resistant allele prevents optimal drive transmission, and if selected for will ultimately remove the drive allele from the population. However, several studies have recently shown that this problem can be mitigated, or overcome completely, by directing the Cas9 nuclease to highly conserved loci, targeting the allele with multiple guide RNAs, and restricting the expression of Cas9 to early germline cells (Noble et al., 2017; Champer et al., 2018, 2020; Kyrou et al., 2018; Carballar-Lejarazú et al., 2020; Hammond et al., 2021).

**FIGURE 1:**
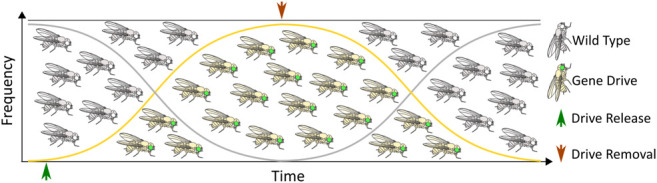
The ideal gene drive: An autonomous homing gene drive designed to spread rapidly through a target population that is also amenable to scar-free excision leading to restoration of wild type alleles.

**FIGURE 2:**
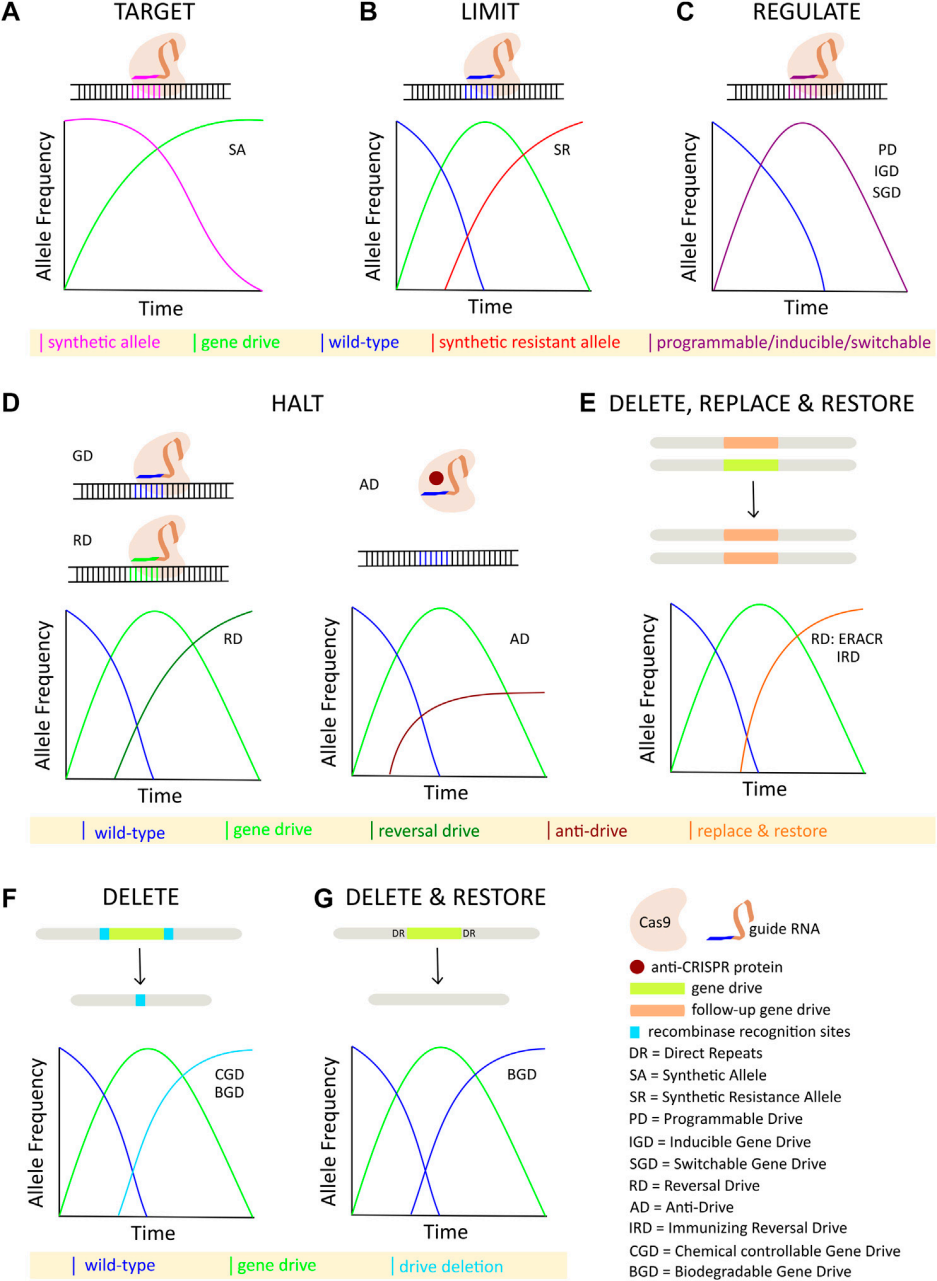
Overview of strategies for controlling CRISPR-based autonomous homing gene drives: **(A)** Synthetic target sites are specifically cleaved and homed into by the autonomous drive element. **(B)** Inundation with synthetic resistant alleles limits the spread of an autonomous drive. **(C)** Titration and/or induction of various programmable, inducible, or switchable components of the autonomous drive regulate its spread. **(D)** Spread of autonomous drive is halted either by a second drive targeting the first drive, or by a protein-based anti-Cas9 interaction. **(E)** Autonomous drive is deleted and replaced with a second element carrying a rescue gene. **(F)** Autonomous drive is deleted by inducing recombination between transgenic target sites flanking the drive allele. **(G)** Autonomous drive is deleted by homology-based intramolecular recombination leading to restoration of the native wild-type alleles.

Although some technical challenges remain, rapid advancements in the design and development of gene drives capable of mitigating the selection of genetic resistance alleles suggests that the development of technologies for limiting the spread of drives through a population will be necessary, if such tools are ever to be successfully tested and deployed in the field. The ideal technology would permit completely efficient genetic drive, but if necessary could be induced to initiate a process ultimately leading to the removal of the drive element, and complete restoration of the population to the original wild-type state (Figure 1). While no such technology currently exists, a number of systems for controlling CRISPR-based autonomous homing gene drives have been proposed, with many having already demonstrated proof-of-principle in model or non-model organisms. While progress in this area has not proceeded as rapidly as the development of the drives themselves, these studies provide valuable insights into the design and implementation of systems for managing the risks inherent to conducting genetic engineering on a massive scale. Thus, we summarize here the current theoretical and technological advancements made in controlling CRISPR-based autonomous homing drives (Figure 2; Table 1), assessing the strengths and weaknesses of each, and where applicable suggesting improvements that may be necessary for the approval and application of these innovative strategies. We have organized this information by organism, where appropriate. Although several designs for split/daisy drives have also been experimentally demonstrated as effective methods for controlling the spread of gene drives through a population, these are not included here as this review instead focuses on the development of technologies that could be incorporated into a single autonomous drive element.

**TABLE 1:** Studies on controlling CRISPR-based autonomous homing gene drives.

Drive control	Acronym	Control mechanism	Intended outcome	Species	References
Synthetic Allele	SA	Sequence polymorphism	Target specific populations of a species	Yeast	DiCarlo et al., (2015)
Reversal Drive	RD	Overwriting Drive	Halt or delete gene drives	Yeast	(Esvelt et al., 2014; DiCarlo et al., 2015)
		ERACRs, e-CHACRs		*Drosophila*	(Gantz and Bier, 2016; Xu et al., 2020)
		CATCHA		*Drosophila*	Wu et al., (2016)
		Cas9 deactivation		Theoretical	(Vella et al., 2017; Girardin et al., 2019; Rode et al., 2020)
Programmable Drive	PD	Cas9 and sgRNA programming	Titrate/regulate drive propagation	Yeast	(Roggenkamp et al., 2018; Goeckel et al., 2019)
Anti-Drive	AD	Anti-CRISPR proteins	Halt gene drive spread	Yeast	Basgall et al., (2018)
				Anopheles	Taxiarchi et al., (2021)
Chemical controllable Gene Drive	CGD	Small molecule-induced “off switch”	Excise gene drive without restoration of wild-type	*Drosophila*	Chae et al., (2020)
Inducible Gene Drive	IGD	Small molecule-induced “on switch”	Spatiotemporal regulation of gene drive activity	*Drosophila*	López Del Amo et al., (2020)
Switchable Gene Drive	SGD	Genetic code expansion	Spatiotemporal regulation of gene drive activity	Mouse	Suzuki et al., (2018)
Synthetic Resistance	SR	Synthetic resistance alleles	Drive extinction through introgressed resistance	Theoretical	(Burt, 2003; Vella et al., 2017; Rode et al., 2019)
Immunizing Reversal Drive	IRD	Recoded functional gene	Replace initial gene drive and wild type with a second drive carrying a functional recoded allele	Theoretical	(Esvelt et al., 2014; Vella et al., 2017; Rode et al., 2020)
Biodegradable Gene Drive	BGD	Self-elimination	Excise gene drive with or without restoration of wild-type	Theoretical	Zapletal et al., (2021)

### 1.1 Yeast

#### 1.1.1 Synthetic Allele

Various systems for controlling CRISPR-based autonomous homing gene drives have been demonstrated in the unicellular eukaryotic model organism, *Saccharomyces cerevisiae*. One such system involves the introgression of a neutral synthetic DNA sequence into a natural population prior to the deployment of an autonomous drive. The drive is then programmed to target the introduced sequence, thereby restricting its spread only to the genetically modified organisms. This strategy establishes an effective barrier against unwanted genetic drive into non-target organisms or neighboring populations of the same species. In proof-of-principle studies, gene drive was observed in cells bearing the synthetic sequence at frequencies >99%, and not at all in yeast with wild-type sequences (DiCarlo et al., 2015). This strategy has some attractive features, and is relatively trivial to achieve in a laboratory setting. However, genetically modifying natural populations of multicellular organisms prior to drive release would be orders of magnitude more complex, with the introgressed sequences subject to Mendelian rates of inheritance, and very likely undesirable. Thus, this strategy is probably better suited to the confinement of drives in laboratory settings as an effective safeguard against escape. Naturally occurring sequence polymorphisms present in isolated populations might be amenable to such a strategy, so called precision drive; however, similar to HEG target sites these would likely be relatively rare and of limited utility (Esvelt et al., 2014).

#### 1.1.2 Reversal Drive

Follow-up drives have been proposed to be used as “braking systems”, halting and even reversing genome alterations affected by an earlier drive that has already spread through a population. One such system employs what has been termed an “overwriting drive”, which carries a functional copy of the gene disrupted by the initial drive (a rescue gene), a source of Cas9, and a guide RNA that targets the initial drive. In yeast, the overwriting drive replaced the initial drive at an efficiency >99%, in subsequent generations restoring the function of the gene that was initially targeted by the earlier drive (Esvelt et al., 2014; DiCarlo et al., 2015). The overwriting process is confined to drive-bearing organisms, but only replaces one drive with another, leaving behind genetically modified transgenic organisms, which is likely to be undesirable considering the underlying motivation for removing the first drive may well apply to the second one as well.

#### 1.1.3 Programmable Drive

Cas9-based autonomous drives invariably contain a nuclease, Cas9, and guide RNA (sgRNA). Thus, the expression, localization, or other properties of these components can be programmed to modulate the transmission rates of the drive element. Multiple independent mechanisms have been demonstrated to be capable of modulating drive transmission, ranging from complete efficiency to no activity. These mechanisms include: 1) titrating the level of Cas9 by controlling expression from the promoter 2) modifying the nuclear localization of Cas9 by including various combinations of nuclear export signals and nuclear localization signals 3) altering the efficiency of Cas9 targeting by varying the length or number of mismatches present in the sgRNA 4) generating tandem fusions with the *S. pyogenes* Cas9 and an enzymatically inoperative or dead dCas9 variant that serves as a competitor in binding an sgRNA (Roggenkamp et al., 2018; Goeckel et al., 2019). The platform developed in yeast provides a system for rapidly testing various tunable aspects of, or evaluating new methods for programming the performance of, gene drive constructs. The discoveries made in yeast, however, will need to be translated to sexually reproducing multicellular organisms. Further, as with most of the strategies summarized here, each of these mechanisms would need to achieve a “Goldilocks” zone of activity, where the gene drive is not hampered enough to prevent the achievement of population control or replacement over a specified period of time before ultimately being inactivated, which in practice might be difficult. Also similar with most of the other strategies, transgenic populations would be likely to persist for an extended period of time, unless the drive allele was associated with a significant fitness cost.

#### 1.1.4 Anti-Drive

Naturally occurring anti-CRISPR (Acr) inhibitor proteins have been identified in several bacteriophages (Dong et al., 2017; Rauch et al., 2017; Liu et al., 2019). Acr proteins are DNA mimics that directly interact with the Cas9 protein to competitively inhibit binding with the sgRNA-guide sequence and subsequent Cas9-mediated cleavage. In a haploid yeast model, gene drive homing was almost completely inhibited by the anti-CRISPR peptides, AcrIIA2 and AcrIIA4 (Basgall et al., 2018). Integration of these peptides into the gene drive construct under inducible promoters permitted drive inhibition to be titrated to levels that were lower than the nearly complete abrogation observed previously. However, the inducible promoter used did exhibit leaky expression of the AcrIIA2/AcrIIA4 transcripts, which has been commonly observed in most if not all inducible systems tested. Further, it is not clear how such a system might be implemented outside of a highly controlled laboratory environment. An earlier study demonstrated that the activity of these proteins is sensitive to the temporal and spatial presence of the inhibitor (Shin et al., 2017).

Intriguingly, mutational scanning revealed several amino acid positions that resulted in a partial loss of inhibitory activity, suggesting another way that drive inhibition might be titrated (Basgall et al., 2018). Such strategies would face the same challenges as other tunable systems, which are described above. Anti-drive systems indeed have great potential in halting the unwarranted spread of Cas9-based autonomous homing gene drives, but will need to be evaluated further in sexually reproducing diploid organisms.

### 1.2 *Drosophila*

#### 1.2.1 Reversal Drive

Several reversal drives have been developed in the model organism, *Drosophila melanogaster*. These drives have also been referred to as braking systems. Braking systems are based on various designs of transgenic genetic elements that carry one or more sgRNAs, but are devoid of any source of Cas9. These elements are typically designed to target the Cas9 sequence within an autonomous drive that has already spread through a population. The sgRNA or sgRNAs derived from the braking element associate with the Cas9 protein produced from the drive allele, either mutating the Cas9 sequence, or replacing the drive allele with that of the reversal element (Gantz and Bier, 2016; Wu et al., 2016; Xu et al., 2020).

For example, in the eCHACR (erasing construct hitchhiking on the autocatalytic chain reaction) iteration, a neutralizing element is inserted into the genome at a location independent of the drive allele, but is capable of self-copying and inactivating the Cas9 sequence of the gene drive. The eCHACR construct functions by exploiting the tendency of the Cas9 nuclease to generate alleles resistant to further cutting, resulting from erroneous repair of the double stranded break through non-homologous end joining (NHEJ) (Xu et al., 2020). The eCHACR concept was demonstrated to be capable of neutralizing a gene drive in multi-generation cage trials. However, eCHACRs do not delete the original gene drive element. Additionally, the neutralizing element itself introduces an additional transgene into the population. In a similar approach, a CATCHA (Cas9-triggered chain ablation) transgene is inserted directly into the Cas9 sequence of the drive element, inactivating the nuclease (Wu et al., 2016). In the eCHACR and CATCHA technologies, the genetic element may encode one or more sgRNAs. These sgRNAs may be directed at the insertion site to facilitate gene conversion and self-copying, or at the Cas9 sequence of the gene drive allele in order to inactivate it. In another design, an ERACR (element reversing the autocatalytic chain reaction) self-copying element is inserted directly into a locus in the drive allele, thereby deleting and replacing it (Xu et al., 2020). ERACRs encode two different sgRNAs, but they are directed at target sites flanking the drive element to facilitate its exchange with the ERACR transgene. ERACR elements may be engineered to carry an in-frame re-coded portion of the disrupted target gene, restoring its function. In multi-generation population studies, ERACRs were found to efficiently replace a gene drive. However, in some cases the ERACR element produced unexpected recombination events, damaging the target chromosome, and causing negative fitness effects (Xu et al., 2020). In other cases, the gene drive remained, again through the generation of alleles resistant to further cutting by the Cas9 nuclease. In each of these designs, the neutralizing elements encode sgRNAs, but rely on the Cas9 from the gene drive to act *in trans*, encoding none of their own.

#### 1.2.2 Chemical controllable Gene Drive

The spread of an autonomous homing gene drive can be controlled by incorporating a chemically responsive “off switch”. Such a chemically controllable gene drive (CGD) cassette has been attempted with the following components: 1) an autonomous homing gene drive element 2) a site-specific recombinase (Rippase) driven by a modified GAL4/UAS system, activated by RU486 (GeneSwitch). The CGD cassette is flanked by recognition sites for Rippase, which aid in the removal of the entire cassette via recombination (Chae et al., 2020). However, in population studies the CGD system was only minimally effective, with the gene drive being eliminated relatively inefficiently. The authors of the study identified several limitations of their experimental design, and acknowledged the need for higher statistical power and optimization of the RU486 effect. In addition, some components of the system are unlikely to be feasible outside of a laboratory environment.

#### 1.2.3 Inducible Gene Drive

An alternative to an “off switch” is an “on switch,” where Cas9-based gene drive activity can be induced by a small molecule. One such inducible gene drive (IGD) system was described in a non-autonomous split gene drive system, where the Cas9 and sgRNAs are encoded in separate loci on the same or different chromosomes (CopyCat drive system). In this system, the Cas9 protein is fused to an unstable protein domain (dihydrofolate reductase (DHFR) from *Escherichia coli*) that is targeted for proteasomal degradation upon expression (DD-*Sp*Cas9). Addition of a small molecule ligand Trimethoprim (TMP) stabilizes DD-*Sp*Cas9, enabling CopyCat drive activity. In a proof-of-principle study, addition of TMP stabilized the Cas9 fusion protein and facilitated a dose-dependent super-Mendelian inheritance of both the DD-*Sp*Cas9 and sgRNA constructs (López Del Amo et al., 2020). Differential homing efficiencies were observed for sgRNA constructs targeting the *white* and *ebony* genes, with the *white* CopyCat drive system producing relatively higher homing rates with increasing doses of TMP. The inducibility of IGDs facilitates spatiotemporal control, which is a highly desirable quality in the development of gene drive technologies. Similar to the CGD “off switch”, and other inducible systems, IGDs also suffer from leakiness, which has proven to be a universal technical challenge in developing any ligand-based system of control. If the leakiness that seems to be an inherent feature of nearly all inducible systems evaluated so far can be addressed, IGDs should be thoroughly tested in autonomous homing gene drive systems, as split configuration drives, such as the CopyCat system, cannot function as low-threshold gene drives when released into natural populations.

### 1.3 Mosquito

#### 1.3.1 Anti-Drive

In the human malaria vector, *Anopheles gambiae*, highly efficient Cas9-based autonomous suppression drives have been developed and evaluated (Kyrou et al., 2018; Hammond et al., 2021). Suppression drives typically target genes that are functionally constrained and cause recessive female sterility, reducing fecundity and resulting in population suppression. As a countermeasure to these highly efficient drives, an anti-drive system was developed consisting of the anti-CRISPR peptide, AcrIIA4. The AcrIIA4-based anti-drive system inhibited homing of two different Cas9-based suppression gene drives targeting either the fertility gene, AGAP007280 (*Drosophila* orthologue of *nudel*) or the sex determination gene, *doublesex*. In cage trials, introgression of anti-drive males prevented population suppression by the drive targeting *doublesex* (Taxiarchi et al., 2021).

Unlike reversal drives that rely on nuclease-based cutting of DNA, anti-drive systems have not been associated with unintended genome alterations at or near the drive target site, as they are premised on protein-protein interactions. Anti-drive systems that are not incorporated into the gene drive construct will be subject to normal rates of Mendelian inheritance, as they do not include any homing mechanism. Thus, the persistence of these constructs in a population is determined by the relative fitness associated with the anti-drive element. Anti-drive alleles associated with relatively low fitness costs may persist in populations, establishing a general barrier to any new introductions of CRISPR-based gene drives, which may or may not be a desirable feature (Taxiarchi et al., 2021). Incorporation of an inducible anti-drive system into an autonomous homing gene drive construct might permit spatiotemporal titration of drive transmission, but would also need to overcome all of the challenges associated with other inducible systems (elaborated on above).

### 1.4 Mouse

#### 1.4.1 Switchable Gene Drive

Inducible systems that employ small molecules, such as RU486 and Trimethoprim, often exhibit leakiness, which presents problems for applications in regulating gene drives (Chae et al., 2020; López Del Amo et al., 2020). Further, while many of these systems are commonly used in a laboratory setting, environmental use would be both unsafe and impractical. For example, RU486 or mifepristone, is a component of a drug combination often used to induce abortions. Genetic code expansion, which is widely used in synthetic biology, has been proposed as a method for generating Cas9 variants that could function as part of an alternative system for achieving non-leaky control over the expression of the nuclease (Suzuki et al., 2018; de la Torre and Chin, 2021). Such Cas9 variants contain one or more modified codons that incorporate non-canonical amino acids (ncAAs) only in the presence of the corresponding orthogonal aminoacyl-tRNA synthetase/tRNA (aaRS/tRNA) pairs. For example, lysine codons within the Cas9 sequence were replaced with an amber stop codon (UAG) to produce a truncated non-functional protein. However, in the presence of an orthogonal aaRS/tRNA pair that recognizes and incorporates an ncAA, H-Lys (Boc)-OH (BOC), at the codon, a full-length functional Cas9 protein was produced. Expression of the BOC-inducible Cas9 (Cas9^BOC^) was not leaky, and capable of editing both a reporter sequence (*eGFP*) and two different endogenous genes (*Sry* and *Tyr*) in mouse embryos. However, editing of the reporter gene was somewhat limited with either Cas9 or the Cas9^BOC^, presumably due to position effects (Suzuki et al., 2018). Nevertheless, the study demonstrates that ncAA-mediated control of Cas9 expression, through expansion of the genetic code, can be a valuable tool in developing stringent control over Cas9-based gene drives. However, relatively extensive genome engineering will be required to generate organisms with the essential components.

### 1.5 Theoretical Models

#### 1.5.1 Synthetic Resistance

Resistant alleles have the potential to be strongly selected for when insertion of an autonomous homing drive is associated with a highly deleterious phenotype. Thus, the introduction of organisms with target genes possessing fully functional resistant alleles could act as a braking system that is capable of extinguishing a gene drive. This approach is predicted to be more effective against drives that are associated with high fitness costs and large genetic loads (i.e., suppression drives), but less effective against drives that are associated with low fitness costs (i.e., modification/replacement drives). This is because natural selection will strongly favor functional, but drive-resistant, alleles with little to no fitness cost over drive alleles with higher fitness costs (Burt, 2003; Vella et al., 2017; Rode et al., 2019). Deterministic models indicate that to effectively remediate a drive-containing population the introduction of synthetic resistance alleles would need to achieve high thresholds in order to counter ongoing conversion of wild-type alleles to gene drive alleles (Vella et al., 2017).

#### 1.5.2 Reversal Drive

As reversal drives are designed to target Cas9 sequences within autonomous drives, they are not predicted to affect wild-type alleles. Deterministic models indicate that at high release frequencies reversal drives will rapidly establish an equilibrium with wild-type alleles. However, at low release frequencies an initial increase in drive alleles will subsequently be followed with an increase in reversal drive alleles, ultimately reducing the frequency of drive alleles in the population (Vella et al., 2017). Other deterministic models that view drive/brake/wild type alleles in a rock/paper/scissors scenario find that coextinction of the drive/brake alleles is only possible when the fitness costs associated with these alleles are small enough (Girardin et al., 2019). Stochastic models of autonomous suppression drives that consider variable population size, but not the evolution of resistant alleles, indicate that braking systems might only be effective with threshold-dependent drives (Rode et al., 2020).

#### 1.5.3 Immunizing Reversal Drive

Immunizing reversal drives are designed to recall unwanted drives while restoring gene function. Immunizing reversal drives typically carry multiple guide RNAs, their own source of Cas9, and a re-coded copy of the wild-type gene. Immunizing reversal drives target both the unwanted initial drive and wild-type alleles. The re-coded functional gene copy not only protects the IRD against being targeted by the initial drive (immunizing), but also restores normal gene function (reversal) (Esvelt et al., 2014). Deterministic models indicate that IRDs rapidly reach fixation, irrespective of release size. This is because IRDs replace both the unwanted initial drive and wild-type alleles, and therefore do not coexist with the other alleles in a polymorphic equilibrium (Vella et al., 2017). Stochastic models also recommend IRDs as the preferred braking system, as they restore fitness and are likely to spread through a population more quickly (Rode et al., 2020). Therefore, IRDs might represent the fastest way to counter and replace unwanted drives. However, deterministic modeling of suppression drives that naturally generate resistant alleles indicate that IRD alleles will be associated with relatively lower fitness than these alleles, and would eventually fall out of the population (Vella et al., 2017).

#### 1.5.4 Biodegradable Gene Drive

A biodegradable gene drive (BGD) can be described as an autonomous homing transgene that can be pre-programmed to self-eliminate (*via* intramolecular recombination) after achieving its intended goals. Biodegradable gene drives are composed of an autonomous homing element (e.g., Cas9 nuclease with sgRNA) and a self-eliminating element (a second nuclease or pair of nucleases with one or more target site(s) in the transgene). The nuclease in the self-eliminating element can be a recombinase, integration-deficient transposase, or endonuclease. While recombinase-mediated excision of a BGD would leave behind one of its two target sites, transposon or endonuclease-mediated BGD excisions would result in the restoration of wild-type alleles. Deterministic models of BGDs, simulating suppression drives, indicate that autonomous drive transmission would be rapidly reversed, even with a relatively modest rate of self-elimination (<10%) (Zapletal et al., 2021). As BGDs result in the restoration of wild-type alleles and the removal of transgenes, they may represent a crucial development in addressing legitimate ethical, regulatory, and environmental concerns associated with gene drive technologies. However, at the moment BGDs remain purely theoretical and a number of technical challenges will undoubtedly need to be overcome in order to develop working technologies that can subsequently be investigated in laboratory and field settings. For example, inclusion of self-eliminating activity at the point of drive release may face many of the same challenges as other strategies (elaborated on above). Similarly, while an inducible system that activates the self-eliminating mechanism might be preferable, a system enabling tight spatiotemporal control over autonomous drive transmission in either a laboratory or field setting remains to be identified, tested and optimized.

## 2 Conclusions and Future Directions

CRISPR-based autonomous homing gene drives are an innovative and potentially transformative technology. An important aspect of responsible conduct of research into such a powerful technology is the development of countermeasures that can if required halt or reverse any unwanted or undesired outcomes. These countermeasures should be sufficiently mature before any gene drive technologies are approved for biocontrol, not only in the event of an emergency, but also to potentially recall a drive that has achieved its intended goals.

Nearly all of the controlling systems developed so far are designed to stop and/or reverse the drive, but rarely address the environmental persistence of transgenes. Both empirical studies and modeling of braking systems have found that transgene persistence is dependent on the fitness costs associated with both the drives being targeted and the braking systems themselves. With regard to reversal drives, IRDs are predicted to be the most efficient braking systems; however, relatively high fitness costs may cause them to fall out of a population (Vella et al., 2017). Further, immunizing reversal drives have not yet been extensively tested in laboratory settings, so modeling results still need to be confirmed with *in vivo* studies. Several other types of reversal drives have been developed and tested, both empirically and theoretically. While these might be useful in emergencies, to halt the spread of an unwanted drive, evaluation of these systems is far from complete. Large-scale laboratory experiments designed to evaluate the ability of braking systems to stop or replace a gene drive have revealed unintended genetic outcomes that included the introduction of substantial fitness costs and the persistence of transgenes retaining full or partial drive potential. In this regard, anti-drive systems that halt drive activity through protein-protein interactions might be preferable, as these systems have not been associated with unintended genome alterations, but similarly require further testing and modeling under various scenarios. Biodegradable gene drives incorporating self-elimination mechanisms also might be a viable alternative, with the potential to directly address transgene persistence through mechanisms capable of replacing drive alleles with wild-type alleles. However, at the moment, BGDs are largely theoretical, requiring extensive research and development.

A number of gene drive strategies currently under development are based on the premise of population suppression. In these scenarios, a drive allele would introduce a fitness load or gender bias into a target pest species. However, the enormous selection pressures that will be brought to bear on these species from these gene drive-based approaches would be likely to strongly select for any resistant genotypes that might be generated through genetic mechanisms, which are difficult to predict or control. Thus, the eventual outcomes of such approaches may be similar to that of the DDT-based Global Malaria Eradication Program, which was initially very successful, but ultimately resulted in the development of widespread DDT resistance in mosquito populations. Spatially and temporally limiting gene drives might prevent the evolution of resistance to gene drive strategies by reducing these selection pressures. While such an approach would likely require multiple releases, this might be preferable to the generation of resistance. The evolution of resistance to gene drive activity might not be limited to the presence or generation of fitness costs or unexpected genomic events. Naturally occurring selfish genetic elements (i.e., transposons) are a constant source of genetic conflict and subject to control by genomic defenses (reviewed in Werren et al., 1988; Kazazian, 2004; McLaughlin and Malik, 2017). Extensive and widespread use of synthetic Cas9-based gene drives may ultimately result in similar genetic conflicts, leading to targeting by evolutionarily conserved small RNA pathways, such as those generating PIWI interacting RNAs (piRNAs) and short interfering small RNAs (siRNAs), which might be difficult to circumvent. Finally, it seems unlikely that any current technology can adequately address all of the concerns associated with the use of gene drives. Therefore, it is important that many different control strategies continue to be investigated and considered.
